# Effect of Macro Fibers on the Permeability and Crack Surface Topography of Layered Fiber Reinforced Concrete

**DOI:** 10.3390/ma17081733

**Published:** 2024-04-10

**Authors:** Wei Zeng, Weiqi Wang, Qiannan Wang, Mengya Li, Lining Zhang, Yunyun Tong

**Affiliations:** 1 School of Civil Engineering and Architecture, Zhejiang University of Science and Technology, Hangzhou 310023, China; wei-zeng@zust.edu.cn (W.Z.); 121032@zust.edu.cn (W.W.); wangqiannan@zust.edu.cn (Q.W.); 2 Zhejiang International Science and Technology Cooperation Base for Waste Resource Recycling and Low-Carbon Building Materials Technology, Zhejiang University of Science and Technology, Hangzhou 310023, China; 3 Zhejiang Construction Investment Group Co., Ltd., Hangzhou 310013, China; 4 L2MGC, EA 4114, CY Cergy Paris Université, 95000 Cergy, France; mengya.li1@cyu.fr; 5 Department of Mechanical and Mechatronics Engineering, The University of Auckland, Auckland 1010, New Zealand; lzha977@aucklanduni.ac.nz

**Keywords:** permeability, crack surface topography, layered fiber-reinforced concrete, layering ratio, uniaxial tensile load

## Abstract

This paper describes the effects of macro fibers on permeability and crack surface topography of layered fiber-reinforced concrete (FRC) specimens with different layering ratios under uniaxial tensile load. The crack permeability of layered FRC specimens is investigated by a self-designed permeability setup. The topographical analysis of crack surfaces is investigated by a custom-designed laser scanning setup. The results show that when the fiber volume content and layering ratio of the FRC layer are constant, the tensile toughness of layered FRC specimens depends on the proportion of steel fiber in macro fibers, and with an increase in the proportion of steel fiber, the tensile toughness of layered FRC specimens increases. For the layered FRC specimens, the crack permeability is much lower than that of the normal concrete (NC) specimen. A significant positive synergistic effect on crack impermeability can be achieved by the combination of steel fiber and polypropylene fiber in the SF80PP2.3 specimen. The crack surface roughness parameter (*R*_n_) values of the NC layer in layered FRC specimens are all higher than those of the NC specimen, and the crack surface *R*_n_ of the FRC layer in layered FRC specimens is higher than that of the unlayered FRC specimens. This can effectively increase the head loss of cracks and reduce the crack permeability of layered FRC specimens.

## 1. Introduction

The primary pathways for water penetration in concrete include the concrete matrix and the concrete crack [1,2]. The mechanical strength and density of sound concrete correlate with its impermeability [3]. The utilization of fibers increases the internal interfaces of concrete, and this results in a reduction in the compactness and an increase in the permeability of the concrete matrix [4]. The crack surface of fiber-reinforced concrete (FRC) is rougher than that of normal concrete (NC). The rough crack surface of concrete increases the hydraulic head loss [5]. Simultaneously, fibers restrict the propagation of cracks in concrete and transform macrocracks into microcracks. This leads to a reduction in the width of cracks and decreases the crack permeability of concrete [4,6,7,8].

Due to the low tensile strength of the concrete, concrete structures work with cracks in the service stage [9,10,11,12]. The existence of the cracks significantly increases the permeability of the concrete [13]. Akhavan et al. [14] found that the crack permeability of concrete (the effective crack width ≤ 200 μm) is about 1 × 10^−16^ m^2^ to 1 × 10^−9^ m^2^, and the permeability of the concrete matrix is about 1 × 10^−17^ m^2^. Hosseinzadeh et al. [15] found that macro fibers can increase the roughness of the crack surface and reduce the crack permeability of concrete. Thus, the use of FRC can reduce the crack permeability of concrete structures and simultaneously enhance their load-bearing capacity. Additionally, in practical engineering, the cross-sectional area of cracks on the surface of concrete structures is much smaller than the overall surface area of the concrete matrix in concrete structures. The permeability of the concrete matrix cannot be overlooked. Compared to FRC specimens, the NC specimen has an advantage in terms of the impermeability of the concrete matrix, and FRC can significantly decrease the crack permeability of concrete structures [14,16,17]. Therefore, the layered FRC specimens take advantage of the high impermeability of the concrete matrix of NC while also utilizing FRC to increase the crack impermeability. Therefore, the application of the NC layer in layered FRC specimens can also reduce fiber usage, lower construction costs, and improve economic efficiency. From the literature review, we found that there is still a lack of systematic investigation of the layered combination of NC and FRC as layered FRC specimen effect on the permeability and crack surface topography of concrete structures.

In previous studies on the permeability of cracked concrete, cracks were induced by loading, and the permeability was measured after unloading [14,16,18]. However, in practical concrete structures, concrete works under loading, and the crack morphology under load significantly differs from that of the crack after unloading. Consequently, the permeability of cracked concrete after unloading differs from that under loading. In order to replicate real-world conditions in concrete structures, a self-developed permeability setup under axial tensile loads was used to measure the permeability of cracked concrete under loading in real-time. Therefore, the flow of water entering the concrete specimens can be measured to improve the accuracy of the permeability setup for cracked concrete under load.

From the literature review, it was evident that, compared to FRC, NC can effectively enhance the impermeability of the concrete matrix. In contrast, FRC can significantly improve the impermeability of cracked concrete. Therefore, the study investigates the permeability performance trends of layered FRC specimens under load. Additionally, the crack surface roughness of layered FRC specimens was measured to analyse the permeability of cracked concrete. The effects of macro fiber types (including steel fiber, polypropylene fiber, and hybrid fiber (steel fiber + polypropylene fiber)) and layering ratios on the crack permeability of layered FRC specimens were studied. A series of tests were conducted: (a) steel fiber, polypropylene fiber, and hybrid fiber were applied in the FRC, and the layered FRC specimens with different layering ratios were cast; (b) a self-designed vacuum permeability setup was employed in the permeability test to evaluate the crack permeability of the layered FRC specimens under uniaxial tensile load; (c) a custom-designed laser scanning setup was employed to obtain and reconstruct data on the crack surface morphology of different layered FRC specimens. Furthermore, the tensile toughness of layered FRC specimens under a uniaxial tensile load was analyzed. The permeability parameter *α* was employed to analyze the permeability of layered FRC specimens. The roughness parameter was applied to analyze the crack surface topography of layered FRC specimens. This study can provide theoretical data for the application of layered FRC in concrete projects with impermeability and crack resistance requirements.

## 2. Experiment

### 2.1. Materials

The study focused on concrete reinforced with fibers for use in roads, tunnels, and other infrastructure projects. The self-consolidating concrete of C40 strength class with excellent workability was used as the concrete matrix and designed according to the code JGJ/T283:2012 [19]. The materials and the base mix proportion of the concrete matrix are presented in Table 1.

**Figure 1:**
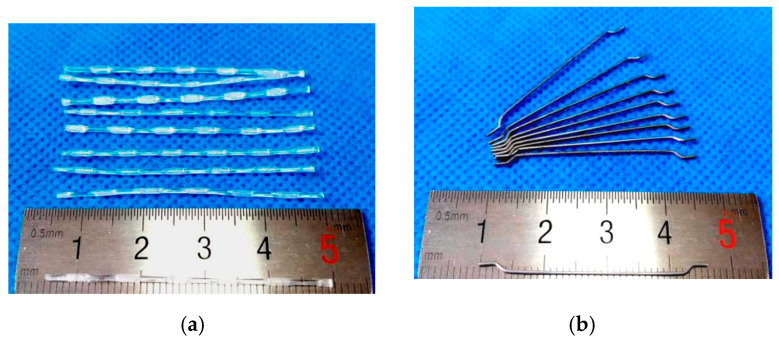
Macro fibers (**a**) polypropylene fiber; (**b**) steel fiber.

Generally, it is referred to as macro fiber when the fiber length exceeds 3 cm. Macro fibers are utilized to enhance flexural toughness, mitigate and bridge macro cracks, and improve residual behavior under load [20]. Previous studies have found that steel fibers with a high elastic modulus and polypropylene fibers with a low elastic modulus exhibit a positive synergistic effect on the load-bearing capacity [21,22]. Therefore, macro-steel fibers and macro-polypropylene fibers were used in this study. The types and properties of fibers are presented in Figure 1 and Table 2. When the fiber content is low, the improvement in the mechanical properties of concrete is limited. However, when the fiber content is high, the workability of fresh concrete is significantly reduced, especially with metal fibers. Based on previous studies [23,24], it was found that when the fiber content is 1.25 vol.%, it can effectively ensure good workability and significantly improve the load-bearing capacity of concrete. Therefore, in this study, the fiber volume content of the FRC layer was 1.25 vol.%. The combination of steel fiber and polypropylene fiber was also considered. The NC specimen (without macro fibers) was the reference specimen. The fiber content, slump, and air content results of fresh FRC are shown in Table 3.

### 2.2. Specimens

**Figure 2:**
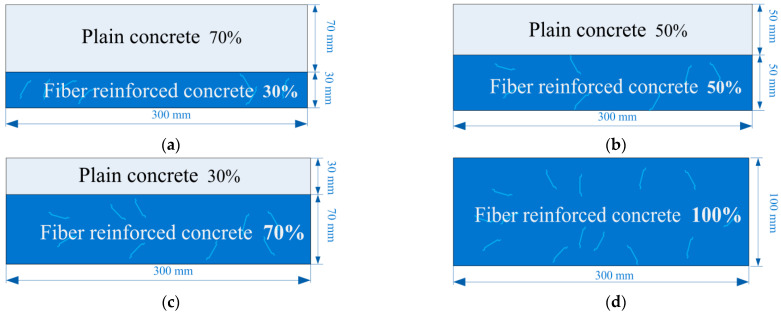
Schematic views of layered FRC specimens with different layering ratios: (**a**) 30%; (**b**) 50%; (**c**) 70%; (**d**) 100%.

In this study, the size of each concrete specimen is 100 mm × 100 mm × 300 mm. Each layered FRC specimen includes the NC layer and the FRC layer. Specimens are classified by the layering ratio (the thickness of the FRC layer relative to the total 100 mm specimen thickness) into categories of 30%, 50%, 70%, and 100% (which are unlayered FRC specimens), as shown in Figure 2. Specimens are named by the fiber type, the fiber content, and the layering ratio of the FRC layer. For instance, a specimen of SF100 with a layering ratio of 30% is called “SF100-30%”.

**Figure 3:**
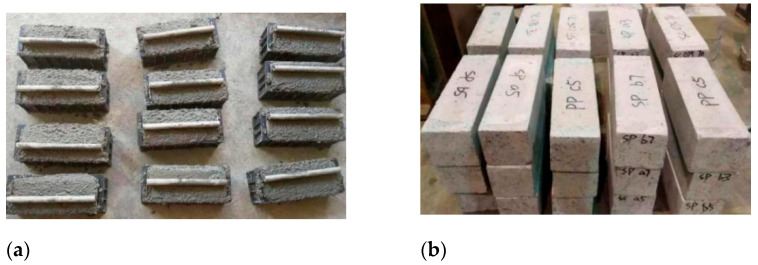
Preparation of layered FRC specimens: (**a**) fresh concrete; (**b**) hardened concrete.

In order to cast the layered FRC specimens, two forced mixers were simultaneously used to mix NC and FRC materials. The fresh concrete from NC and FRC was poured into a concrete mold and divided by a steel plate. A vibrating table was used to remove internal air pores from the fresh concrete. After the fresh concrete was vibrated for 20 s, the steel plate was slowly and vertically removed from the concrete mold during vibration; the total vibration time of the fresh concrete was 30 s. The surfaces of the specimens were covered with plastic wrap to prevent moisture from evaporating. After curing at room temperature (20 °C) for one day, the concrete mold was removed, and the specimens were cured in a standard curing room (temperature of 20 °C and relative humidity of 95%) for 28 days, as shown in Figure 3.

### 2.3. Permeability Test

**Figure 4:**
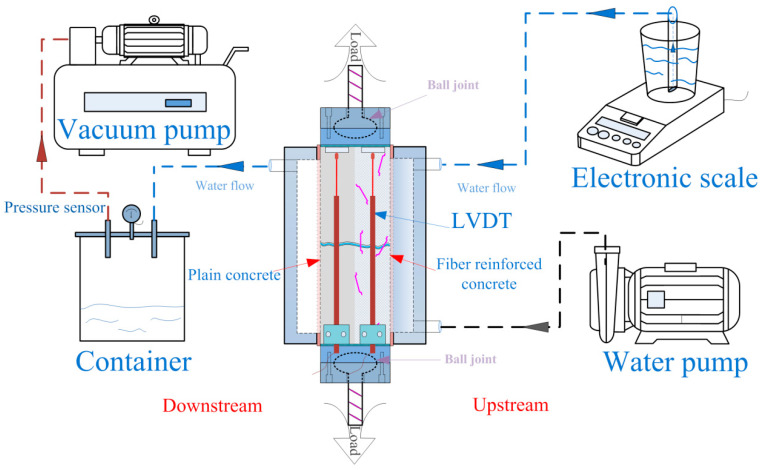
Schematic view of the permeability setup under a uniaxial tension load.

**Figure 5:**
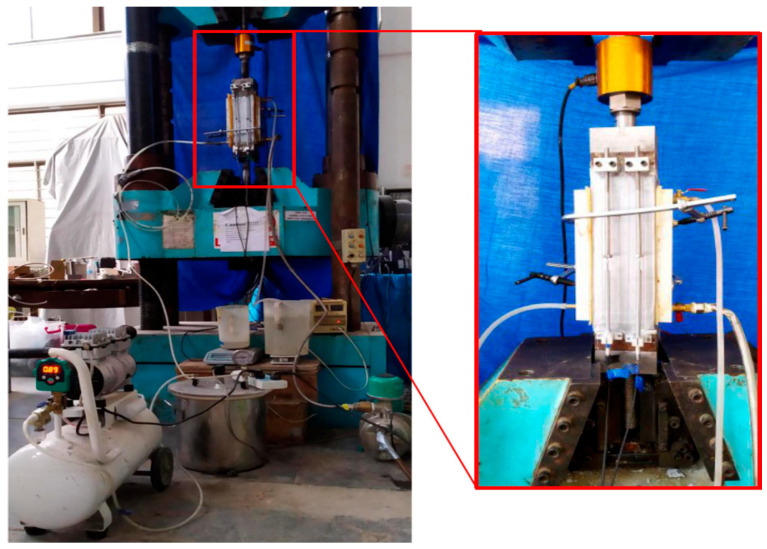
Permeability test of layered FRC specimens under uniaxial tension load.

In order to investigate the permeability of layered FRC specimens under uniaxial tensile load, this experiment utilized a 1000 kN servo-hydraulic testing machine, a vacuum pump, a constant pressure water pump, and an electronic scale, as shown in Figure 4. The operational steps of the permeability test were as follows:

1. Each surface of the specimen was ground to ensure parallelism with its opposite face. Epoxy resin was used to adhere the steel plates to the top and bottom of the specimens. Then, the specimens with steel plates were cured at room temperature of 20 °C for 7 days to ensure the epoxy resin was cured.
2. The vacuum-saturated concrete instrument was used to remove the air from the specimens. The waterproof tape was applied to the side surfaces of the specimens to prevent water leakage.
3. Two bolt clamps were used to fix the water vessels. Rubber rings were employed between the water vessel and the specimens to ensure air tightness. The connection of each instrument is shown in Figure 5.
4. In the experiment, the upstream water vessel was filled with water using a water pump. An upstream rubber tube was inserted into a beaker with water, and an electronic scale measured the mass of water.
5. The vacuum pump maintained an absolute pressure of 10.8 kPa downstream. The upstream was connected to the atmosphere through the beaker, with a pressure difference (Δ*p* = *p*_u_−*p*_d_) of 90 kPa between the upstream and the downstream.
6. In the permeability test, the pressure difference made water break through the specimen. The flow rate and the permeability of the specimen were calculated based on the water reduction rate in the beaker.

In the study, the uniaxial tensile test and permeability test of concrete were carried out simultaneously. Due to the random occurrence and undefined morphology of cracks in concrete under the tensile load, it was impossible to indirectly calculate the seepage cross-sectional area of the cracks in the permeability test, especially when the specimen was fully covered by waterproof tape and water vessels. Therefore, according to previous research [25,26,27], it is necessary to indirectly obtain crack information after cracking. Four LVDTs were used to measure the deflection of specimens under the tensile load in real time. Based on the findings of Seong et al. [28] and Rastiello et al. [13], after cracking, the multiple pieces of concrete can be considered as multiple undamaged elastic blocks. Thus, the crack width can be calculated by the load-deflection curves and LVDTs. This can be used to estimate the crack permeability of concrete under a uniaxial tensile load.

### 2.4. Crack Permeability Parameter

**Figure 6:**
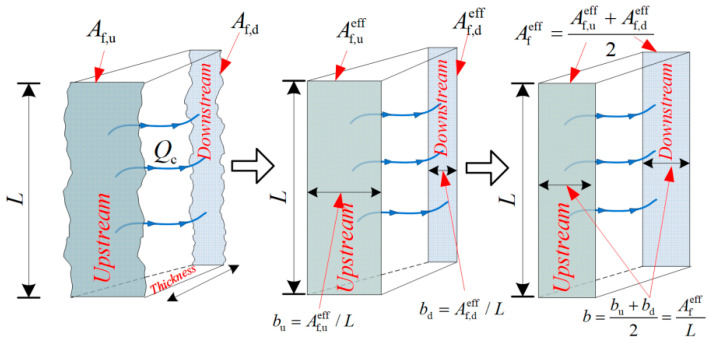
Computational method of effective crack width, where, *A_f_*_,u/d_ represents the crack area on both sides of the specimen, m^2^; *A*^eff^_f/d_ denotes the effective crack area on both sides of the specimen, m^2^; *b*_u/d_ indicates the effective crack width on both sides of the specimen, m.

In the permeability test, the water pressure gradient remained below 1.8 MPa/m, and the Reynolds number was under 1150. This confirmed that the water flow into the concrete specimens maintained a laminar flow. Under the theory of incompressible Newtonian fluid flow, Darcy’s law can be used to assess the crack permeability, and it is calculated by Equation (1).

(1) $$\kappa_{c}=\frac{Q_{c}\mu }{A_{f}^{eff}}{\left(\frac{\Delta p}{\Delta x}\right)}^{-1}=\frac{Q_{c}\mu }{b\cdot L}{\left(\frac{\Delta p}{\Delta x}\right)}^{-1}$$

where, *κ*_c_ represents the concrete crack permeability, m^2^; *Q*_c_ is the water flow rate through the concrete crack, m^3^/s; *µ* is the water dynamic viscosity, 0.001 Pa·s; *ρ* is the density of water at room temperature, 998 kg/m^3^; Δ*p*/Δ*x* is the water pressure gradient, Pa/m; $A_{f}^{eff}$ is the effective area of crack, m^2^; *L* is the length of the crack perpendicular to the flow direction, m; *b* is the effective crack width, m, and the computational method of effective crack width was based on the literature [14], as shown in Figure 6.

When the crack surface is parallel and perfectly smooth, the crack permeability can be calculated by the Poiseuille law, as illustrated in Equation (2).

(2) $$\kappa_{PFM}=\frac{b^{2}}{12}$$

The crack surface in cementitious materials such as mortar, concrete, and rock is rough and irregular. Current research adapts the Poiseuille law for analyzing cement-based material crack permeability by introducing a modified factor *ξ* [13,16,29], which quantifies the effect of surface roughness. The calculation method for this modified factor *ξ* is shown in Equation (3):

(3) $$\kappa_{c}=\xi \frac{b^{2}}{12}$$

Combining Equations (2) and (3), the following is derived:

(4) $$\xi =\frac{\kappa_{c}}{\kappa_{\mathrm{PFM}}}$$

### 2.5. Parameters of Uniaxial Tensile Properties

To evaluate the uniaxial tensile properties of concrete specimens, the parameters of uniaxial tensile properties (*F_P_*, *P*_0.16_, *P*_0.33_, *F*_0.16_, and *F*_0.33_) of FRC specimens are calculated according to Standard CECS13:2009 [30]. *F_P_* is the peak load of the uniaxial tensile load-deflection curve of specimens; *P*_0.16_ is the residual load at an uniaxial deformation of *L*_0_ × 0.16% mm (kN); and *P*_0.33_ is the residual load at an uniaxial deformation of *L*_0_ × 0.33% mm (kN). *F*_0.16_ represents the average bearing capacity of the uniaxial tensile load-deformation curve from 0 to *L*_0_ × 0.16% mm (kN), and *F*_0.33_ is the average bearing capacity of the uniaxial tensile load-deformation curve from 0 to *L*_0_ × 0.33% mm (kN). *P*_0.16_, *P*_0.33_, *F*_0.16_, and *F*_0.33_ can be used to estimate the tensile toughness of the layered FRC specimens. *L*_0_ is the gauge length (*L*_0_ = 300 mm). The calculation methods of *F*_0.16_ and *F*_0.33_ are shown in Equations (5) and (6).

(5) $$F_{0.16}=\frac{\int_{0}^{L_{0}\times 0.16\%}F(x)dx}{L_{0}\times 0.16\%}$$

(6) $$F_{0.33}=\frac{\int_{0}^{L_{0}\times 0.33\%}F(x)dx}{L_{0}\times 0.33\%}$$

### 2.6. Collection of Crack Surface Topography

**Figure 7:**
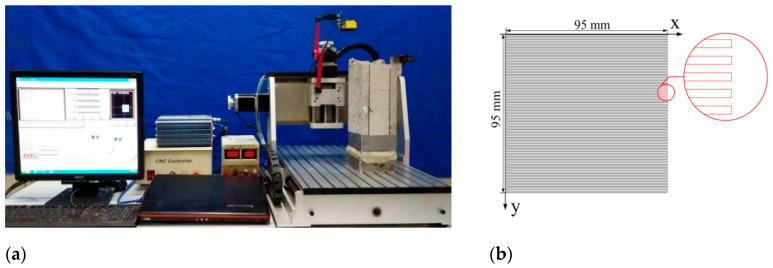
(**a**) Scanning of crack surface of layered FRC specimens; (**b**) Scanning path of crack surface.

After the permeability test, the concrete specimen was divided into two parts along the crack, and the fibers exposed on the crack surface were cut off. A custom-designed laser scanner was developed based on the literature [16] to scan and collect information on the crack surface, as shown in Figure 7a. The scanning area was 95 mm × 95 mm, as shown in Figure 7b. The roughness of the crack surface was evaluated by the crack surface roughness parameter (*R*_n_) [31,32,33,34], which can be expressed in Equation (7).

(7) $$R_{n}=\frac{S_{t}}{S_{o}}$$

where *S*_o_ is the projected area of the scanning region, 95 mm × 95 mm, and *S*_t_ is the area of the crack surface of the specimen, mm^2^.

## 3. Results and Discussion

### 3.1. Uniaxial Tensile Load-Deflection Curve

**Figure 8:**
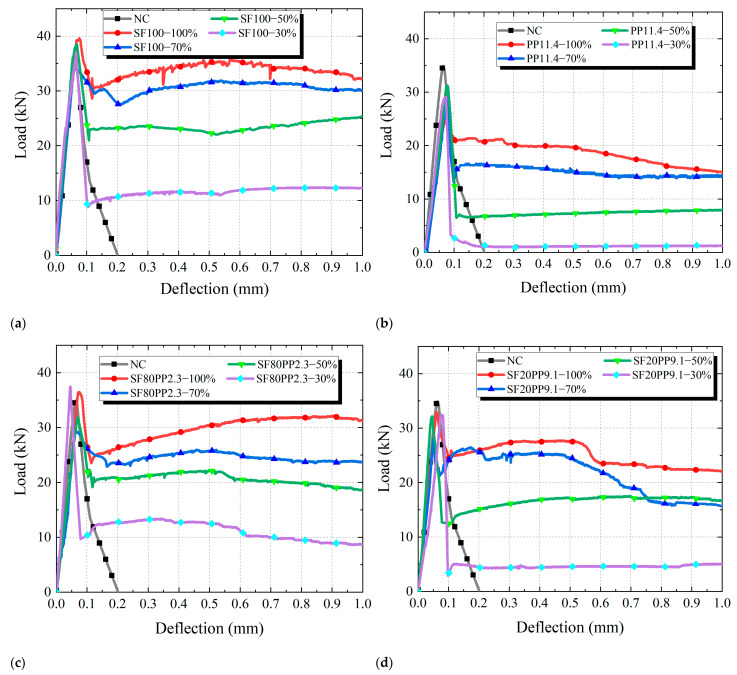
Load-deflection curves of different specimens: (**a**) SF100; (**b**) PP11.4; (**c**) SF80PP2.3; (**d**) SF20PP9.1.

In order to compare the influence of macro fibers on the uniaxial tensile load-bearing capacity of layered FRC specimens, Figure 8 presents a comparison of the load-deflection curves under the uniaxial tensile load of different specimens.

From Figure 8, it can be seen that:

(i) NC exhibits a rapid decrease in uniaxial tensile load-bearing capacity and brittle failure after the peak load. However, the FRC shows a higher level of toughness than the NC.
(ii) For FRC, when the initial cracks form, each group of layered FRC specimens shows a significant reduction in load-bearing capacity, and the reduction in load-bearing capacity decreases with the increasing layering ratios of layered FRC specimens. This can be attributed to that specimens with a high layering ratio have a higher proportion of FRC material and more fibers bridging cracks than those with a low layering ratio, which shows a beneficial effect on the residual strength of concrete.
(iii) Figure 8a–d shows the load-deflection curves of SF100, PP11.4, SF80PP2.3, and SF20PP9.1 specimens at different layering ratios (100%, 70%, 50%, and 30%), respectively. It can be seen that the fiber type has a significant effect on the residual load-bearing capacity after the peak load of the specimen. When the layering ratio of FRC is the same, the load bearing capacity follows the order: SF100 > SF80PP2.3 > SF20PP9.1 > PP11.4. The load bearing capacity of SF100 specimens is higher than that of the other specimens. As the steel fiber content decreases, the residual load-bearing capacity of each group of specimens gradually decreases. This can be attributed to that the bond behavior between steel fibers, which have a high elastic modulus and end hooks, and concrete is significantly higher than that of polypropylene fibers with a low elastic modulus and straight ends. Some studies [35,36] have found similar results.

In order to quantify the uniaxial tensile properties of different specimens, Table 4 compares the parameters of uniaxial tensile properties, including the peak load *F_P_*, the residual loads *P*_0.16_ and *P*_0.33_, and the average bearing capacities *F*_0.16_ and *F*_0.33_.

Table 4 indicates that the *F_P_* values of the SF100, PP11.4, SF80PP2.3, and SF20PP9.1 specimens ranged from 34.3–39.6 kN, 24.6–31.2 kN, 29.5–37.4 kN, and 28.0–32.9 kN, respectively. The SF100 specimen demonstrated slightly higher peak loads than the other specimens. Meanwhile, the order of tensile toughness of layered FRC specimens with the same layering ratio was SF100 > SF80PP2.3 > SF20PP9.1 > PP11.4, except for the *F*_0.16_ values of SF80PP2.3-30% and SF100-30% and the *F*_0.16_ values of SF80PP2.3-70% and SF20PP9.1-70%. It illustrates that when the layering ratio of layered FRC specimens is the same, its tensile toughness is determined by the proportion of steel fiber in macro fibers, and a high proportion of steel fiber results in a high tensile toughness for layered FRC specimens with the same fiber volume content.

To quantify the effect of layering ratio on the load bearing capacity of layered FRC specimens, Table 5 shows the decrease in uniaxial tensile properties of layered FRC specimens compared to unlayered FRC specimens.

Table 5 indicates that with the decrease in layering ratio of layered FRC specimens, the PP11.4 and SF20PP9.1 specimens demonstrate the most notable reduction in the parameters of the uniaxial tensile property. A comparison between the SF100 and SF80PP2.3 specimens reveals that when the layering ratio is 70%, the SF80PP2.3-70% shows a greater relative decrease ratio in the parameters of uniaxial tensile property than those of the SF100-70%. When the layering ratios are between 50% and 30%, the relative decrease ratios in the parameters of the uniaxial tensile property of the SF80PP2.3 specimen are lower than those of both the SF100 and PP11.4 specimens. Consequently, the reduction in the parameters of the uniaxial tensile property of the SF80PP2.3 specimen with hybrid fibers becomes more gradual than those of mono FRC with the decreasing layering ratio.

### 3.2. Crack Permeability of Layered FRC Specimens

**Figure 9:**
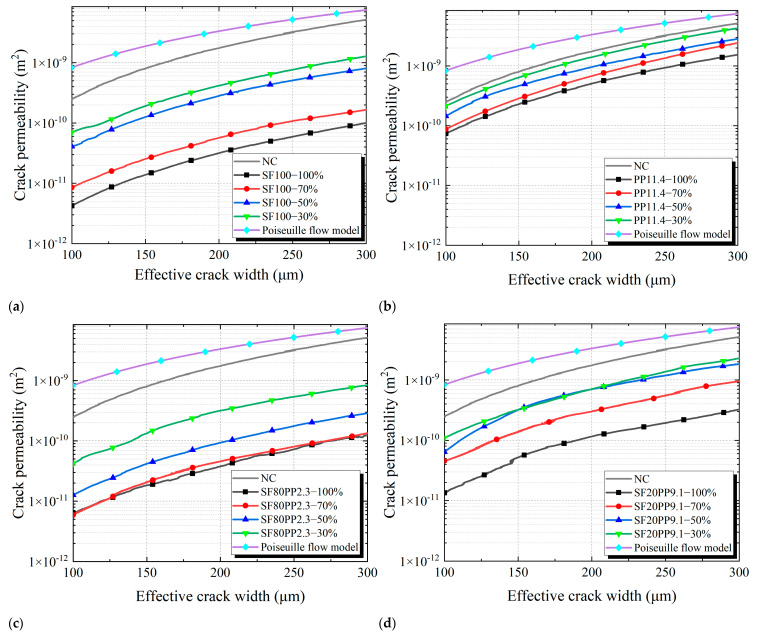
Crack permeability-effective crack width curves of different specimens: (**a**) SF100; (**b**) PP11.4; (**c**) SF80PP2.3; (**d**) SF20PP9.1.

Figure 9 compares the crack permeability-effective crack width curves of different specimens. Due to the stiffness limitation of the servo-hydraulic testing machine, cracks of layered FRC specimens under uniaxial tensile load rapidly expand and propagate, and the crack permeability of small crack widths is difficult to measure. Therefore, the study focuses on the crack permeability of crack widths ranging from 100 to 300 µm. The Poiseuille flow model and the NC specimen serve as reference groups. It can be seen that the difference between the NC and the Poiseuille law is smaller than that between the other layered FRC specimens and the Poiseuille law. This is due to the fact that, compared with FRC specimens, the crack surface of NC specimens is smoother and closer to the Poiseuille law. A similar phenomenon has also been observed in other investigations [16].

Figure 9a–d presents the crack permeability-effective crack width curves of layered FRC specimens with different layering ratios. When the effective crack width is the same, the crack permeability of layered FRC specimens increases as the layering ratios decrease. This is because the thickness of the NC layer of layered FRC specimens increases with decreasing layering ratios. The crack surface area of the NC layer increases and that of the FRC layer decreases in the layered FRC specimens. The crack surface of the NC layer is smoother than that of the FRC layer, and this causes minimal hydraulic head loss. Consequently, the crack permeability of layered FRC specimens increases with the decrease in layering ratios. The crack surface morphology of each group is compared and analyzed in Section 3.3.

Table 6 presents a comparison of the crack permeability of layered FRC specimens with different crack width values (100 μm, 200 μm, and 300 μm).

From Table 6, it can be seen that:

i. With the increase in crack width, the crack permeability of all layered FRC specimens increases.
ii. For mono-FRC with the same layering ratio, the SF100 specimen exhibits lower crack permeability than the PP11.4 specimen.
iii. For hybrid FRC, the crack permeability of the SF20PP9.1 specimen is higher than that of the SF100 specimen and lower than that of the PP11.4 specimen. It indicates that the SF20PP9.1 specimen with hybrid fibers does not exhibit superior crack impermeability performance compared to mono-FRC. However, when the crack width is the same, the crack permeability of the SF80PP2.3 specimen is lower than that of the SF100 and PP11.4 specimens with the same layering ratio. It demonstrates that the SF80PP2.3 specimen with hybrid fibers exhibits greater crack impermeability performance compared to mono-FRC (SF100 and PP11.4) with the same volume of fibers. The SF80PP2.3 specimen demonstrates a positive synergistic effect of hybrid fibers on crack impermeability.

**Figure 10:**
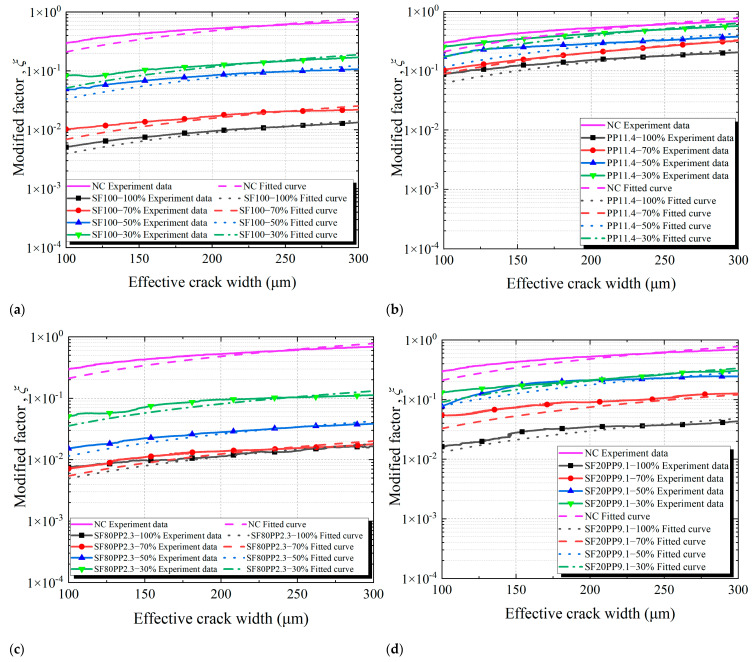
Modified factor *ξ*-effective crack width curves of layered FRC specimens: (**a**) SF100; (**b**) PP11.4; (**c**) SF80PP2.3; (**d**) SF20PP9.1.

The modified factor *ξ* means the ratio of crack permeability to that predicted by the Poiseuille law at a given crack width. The modified factor *ξ* of crack permeability can be calculated by Equation (4). The modified factor *ξ*-effective crack width curves of different layered FRC specimens are shown in Figure 10. 

In previous studies [14,16,29], the modified factor *ξ* was often considered a constant for evaluating crack permeability. However, Figure 10 reveals that with the increment of the effective crack width, the modified factor *ξ* of all samples gradually increases and is not a constant. Consequently, the modified factor *ξ* is unsuitable to characterize the crack permeability. This study utilizes a derivative model of modified factor *ξ* to accurately evaluate the effect of fibers on the crack permeability of concrete specimens. The formula of the model is as follows:

(8) $$\xi =\xi (b)=\alpha b^{\beta }$$

Combining Equations (4) and (8), it can be obtained:

(9) $$\kappa_{c}=\xi \cdot \kappa_{p}=\xi (b)\cdot \kappa_{p}=\alpha b^{\beta }\cdot \kappa_{p}=\frac{\alpha b^{\beta +2}}{12}$$

where *α* represents the crack permeability parameter, b indicates the effective crack width of the specimen, and m.

According to Equation (9), when the effective crack width and parameter *β* are fixed, the crack permeability parameter *α* is directly proportional to the crack permeability *κ*_c_. Therefore, the crack permeability parameter *α* can be used to represent the crack permeability trend within a certain crack width range, so it is more suitable for quantitatively analyzing the effect of macro fibers on concrete crack permeability than the modified factor *ξ*. 

In order to evaluate the influence of macro fibers on the evolution of concrete crack permeability, a linear fitting was performed on the relationships between the modified factor *ξ* and the effective crack width (b) of the concrete specimen based on Equation (9). As suggested by the findings of Rastiello et al. [13], parameter *β* equaled 1.170.

**Figure 11:**
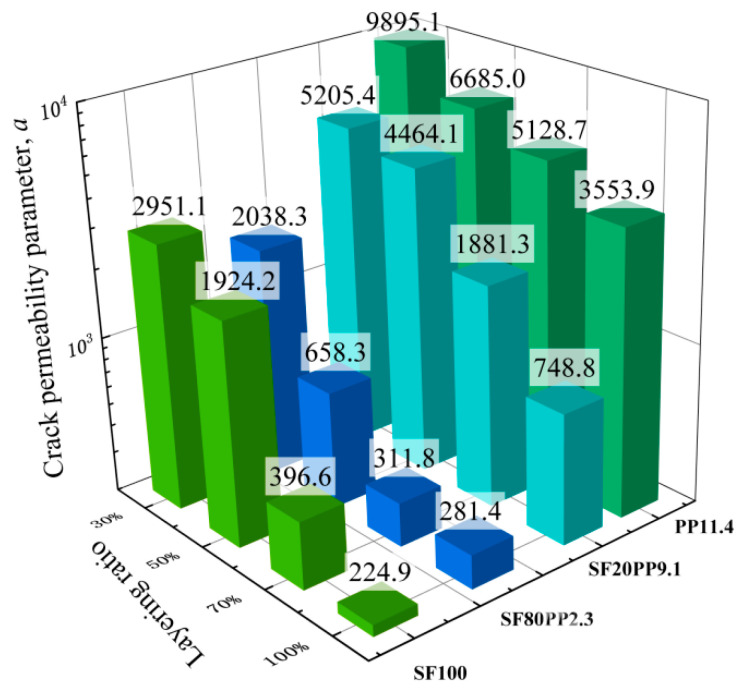
Bar charts of the crack permeability parameter *α* of layered FRC specimens.

Based on Equation (9), the crack permeability parameter *α* can be calculated using the curves of the modified factor *ξ*-effective crack width (b) in Figure 10. The crack permeability parameter *α* remains constant and can serve as a measurement parameter for concrete crack permeability. Figure 11 and Table 7 compare the crack permeability parameter *α* values of different layered FRC specimens.

From Table 7 and Figure 11, it can be seen that:

i. For unlayered FRC specimens (the layering ratio is 100%), the crack permeability parameter *α* follows the order: SF100-100% < SF80PP2.3-100% < SF20PP9.1-100% < PP11.4-100%. The crack permeability parameter *α* value of SF100-100% is the smallest, and this indicates that the crack impermeability of SF100-100% is the best. Compared to SF100-100%, the crack permeability parameter *α* of PP11.4-100%, SF20PP9.1-100%, and SF80PP2.3-100% increased by about 1480.2%, 232.9%, and 25.1%, respectively. This reveals that for specimens with the same fiber volume, an increase in the proportion of steel fiber in macro fibers enhances crack impermeability.
ii. When the layering ratio of layered FRC specimens is 70%, the crack permeability parameter *α* follows the order: SF80PP2.3-70% < SF100-70% < SF20PP9.1-70% < PP11.4-70%. The crack impermeability of SF80PP2.3-70% is the best. Compared to SF80PP2.3-70%, the crack permeability parameter *α* of SF100-70%, SF20PP9.1-70%, and PP11.4-70% increased by about 27.2%, 503.4%, and 1544.9%, respectively.
iii. When the layering ratio of layered FRC specimens is 50%, the crack permeability parameter *α* follows the order: SF80PP2.3-50% < SF100-50% < SF20PP9.1-50% < PP11.4-50%. The crack impermeability of SF80PP2.3-50% is the best. Compared to SF80PP2.3-50%, the crack permeability parameter *α* for SF100-50%, SF20PP9.1-50%, and PP11.4-50% increased by about 192.3%, 578.1%, and 915.5%, respectively. The difference in the crack permeability parameter *α* between SF80PP2.3-50% and SF100-50% becomes obvious.
iv. When the layering ratio of layered FRC specimens is 30%, the crack permeability parameter *α* follows the order: SF80PP2.3-30% < SF100-30% < SF20PP9.1-30% < PP11.4-30%. Among these layered FRC specimens, the crack impermeability of SF80PP2.3-30% is the best. Compared to SF80PP2.3-30%, the crack permeability parameter *α* of SF100-30%, SF20PP9.1-30%, and PP11.4-30% increased 44.8%, 155.4%, and 385.5%, respectively. Compared to layered FRC specimens with a 50% layering ratio, the gap in the crack permeability parameter *α* between SF80PP2.3-30% and other samples with a 30% layering ratio becomes small.

From the discussion above, it is evident that for mono FRC with different layering ratios, steel fiber is more effective in enhancing crack impermeability than polypropylene fiber. For unlayered FRC specimens, the crack permeability parameter *α* of hybrid fiber-reinforced concrete (SF80PP2.3 and SF20PP9.1) specimens falls between the SF100 specimen and the PP11.4 specimen. For layered FRC specimens, the crack permeability parameter *α* of the SF80PP2.3 specimen is lower than that of mono FRC. It indicates that the SF80PP2.3 specimen with hybrid fibers demonstrates a higher crack impermeability than that of mono FRC. When the layering ratio is 50%, the crack impermeability of the SF80PP2.3 specimen is greatest and most significant compared to mono FRC (SF100 and PP11.4).

### 3.3. Crack Surface Roughness

The crack width and crack surface morphology are important factors affecting the permeability of cracked concrete. When the crack width is fixed, a rough crack surface significantly reduces the permeability of the cracked concrete. Consequently, the crack surface roughness of all specimens was measured to analyze the crack permeability performance. Due to the low permeability of the concrete matrix, the permeability of the sound concrete matrix can be disregarded, and the crack permeability of concrete is solely focused [13,14,16]. When the crack width is constant, the rough surface of the concrete crack increases the hydraulic gradient of water flow and decreases the crack permeability of the concrete. This study employed a surface laser scanner to reconstruct the crack surface morphology of different layered FRC specimens.

**Figure 12:**
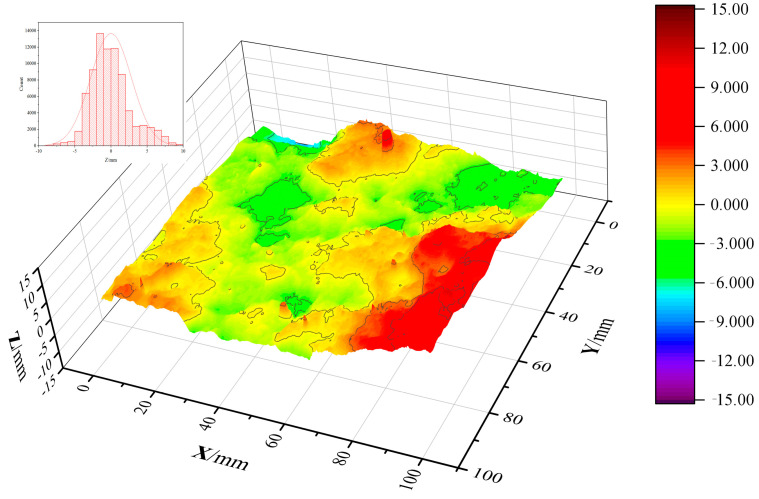
Reconstruction view of the crack surface of the NC specimen.

**Figure 13:**
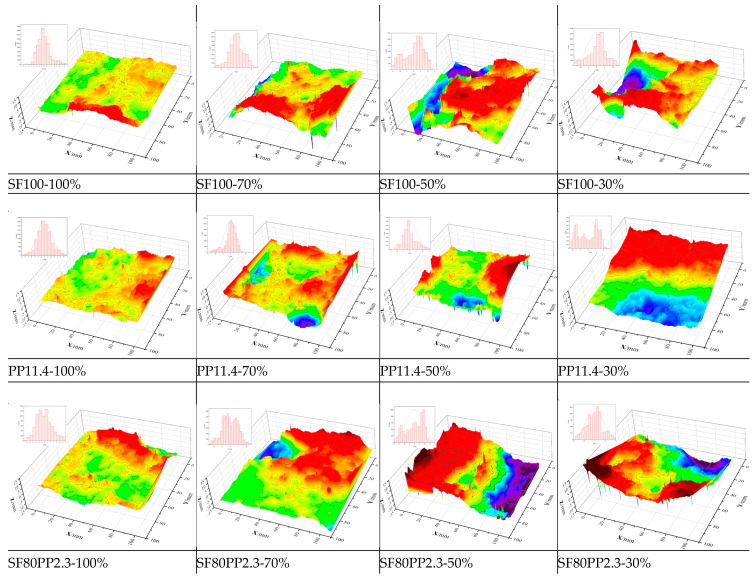

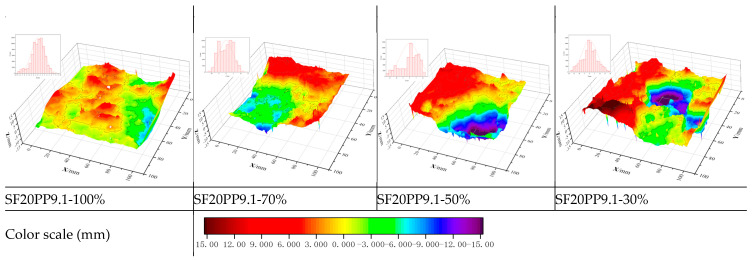
Reconstruction view of the crack surface of layered FRC specimens.

Figure 12 and Figure 13 show the reconstructed crack surfaces of specimens. The color scale of all the reconstructed images is the same. In order to quantify the effect of macro fibers on crack surface characterization, the study employs the *R*_n_ parameter to estimate the crack roughness of the NC layer, the FRC layer, and the entire crack surface of layered FRC specimens. Table 8 compares the crack surface *R*_n_ of different specimens.

From Table 8, it can be seen that:

i. Compared to the layered FRC specimens, the crack surface roughness parameter *R*_n_ of the NC specimen is the lowest. This correlates with its highest crack permeability parameter *α* and the lowest crack impermeability among all specimens.
ii. For unlayered FRC specimens with the same volume content of fiber, the higher the proportion of steel fiber in macro fibers, the higher the *R*_n_ of the entire crack surface. It means that the crack with a high *R*_n_ is rough. For example, the *R*_n_ values of the entire crack surfaces of NC, SF100, PP11.4, SF80PP2.3, and SF20PP9.1 specimens are 1.232, 2.108, 1.796, 2.016, and 1.902, respectively. The SF100 specimen has the highest *R*_n_ value of the entire crack surface. Compared to NC specimens, the *R*_n_ of SF100, PP11.4, SF80PP2.3, and SF20PP9.1 specimens increased by about 71.1%, 45.8%, 63.6%, and 54.4%, respectively.

In order to quantify the effect of layering ratio on the roughness of the entire crack surface of layered FRC specimens, Table 9 shows the decrease rate of *R*_n_ of the layered FRC specimens compared to unlayered FRC specimens.

From Table 9, it can be seen that, compared to unlayered FRC specimens, the *R*_n_ of the crack surfaces of layered FRC specimens decreases progressively with the decreasing layering ratio. For example, for the PP11.4 specimen, the *R*_n_ values of the entire crack surface of PP11.4-100%, PP11.4-70%, PP11.4-50%, and PP11.4-30% are 1.796, 1.658, 1.53, and 1.428, respectively. Compared to PP11.4-100%, the *R*_n_ of the entire crack surface of PP11.4-70%, PP11.4-50%, and PP11.4-30% decreased by about 7.7%, 14.8%, and 20.5%, respectively. With the decreasing layering ratio, the roughness of the entire crack surface of layered FRC specimens decreases. This phenomenon suggests the layered FRC specimens with a low layering ratio show high crack permeability.

In order to quantify the effect of layered FRC specimens with different fiber types and layering ratios on the roughness of the NC layer, Table 10 shows the increase in crack surface *R*_n_ of the NC layer in layered FRC compared to the NC specimen.

From Table 10, it can be seen that the *R*_n_ of the NC layer in layered FRC specimens is higher than the crack surface *R*_n_ of the NC specimen, and the *R*_n_ of the NC layer in layered FRC specimens increases with the increasing layering ratio in layered FRC specimens. For the SF80PP2.3 specimen with hybrid fibers, when the layering ratio is the same, the increase rate of crack surface *R*_n_ of the NC layer is greater than that of mono FRC. For the SF20PP9.1 specimen, the increase rate of crack surface *R*_n_ of the NC layer falls between the SF100 specimen and the PP11.4 specimen, and the PP11.4 specimen exhibits the lowest increase rate of crack surface *R*_n_ of the NC layer compared to NC the specimen. 

Based on previous studies and the analysis above, for layered FRC specimens, the crack surface *R*_n_ of the NC layer is higher than that of the NC specimen and increases with the increment of the layering ratio. Additionally, the higher the content of steel fiber, the rougher the NC layer becomes in the layered FRC specimens. This phenomenon is probably related to the FRC layer in layered concrete. Because the NC layer is closely bonded to the FRC layer, the rough cracks in the FRC layer make the adjacent crack surface of the NC layer rough.

To quantify the effect of layering ratio on the roughness of the FRC layer of layered FRC specimens, Table 11 shows the increase in crack surface *R*_n_ of the FRC layer in layered concrete compared to unlayered FRC specimens.

As shown in Table 11:

i. Compared to unlayered FRC specimens, the FRC layer in layered FRC specimens shows a rough crack surface. For instance, compared to the unlayered FRC specimens with the same fiber type and fiber content, when the layering ratio of layered FRC specimens is 70%, the crack surface *R*_n_ of SF100-70%, PP11.4-70%, SF80PP2.3-70%, and SF20PP9.1-70% increased 1.4%, 0.6%, 2.8%, and -0.4% (decreased by 0.4%), respectively. When the layering ratio of layered FRC specimens is 30%, the crack surface *R*_n_ of the FRC layers of SF100-30%, PP11.4-30%, SF80PP2.3-30%, and SF20PP9.1-30% increased by 2.9%, 3.7%, 5.8%, and 1.6%, respectively. When the layering ratio of layered FRC specimens decreases from 70% to 30%, the crack surface *R*_n_ of the FRC layer of layered FRC specimens increases. The increment in crack surface *R*_n_ of the FRC layer is related to the close arrangement of the two-dimensional randomly distributed fibers in the FRC layer. The thicker the FRC layer of layered FRC specimens is, the closer the distribution of fiber in the FRC layer is to the two-dimensional distribution. It leads to many fibers in the FRC layer being distributed along the direction of stress on the specimen. Many fibers can bridge the cracked surfaces and increase the roughness of the crack surface in the FRC layer of the layered concrete.
ii. Among the layered FRC specimens, the SF80PP2.3 specimen with the different layering ratios shows the most significant increment in crack surface *R*_n_ of the FRC layer compared to that of unlayered FRC specimens. This explains the phenomenon described in Section 3.2: “that the SF80PP2.3 specimen with hybrid fibers has the higher crack impermeability compared to other FRC specimens”. Due to the specific proportions of hybrid fibers and the different elastic moduli of steel fibers and polypropylene fibers in the SF80PP2.3 specimen, it may cause a rougher crack surface than other FRC specimens [37]. Consequently, this contributes to its pronounced impermeability performance.

### 3.4. Crack Topography of the Layered FRC Specimen

**Figure 14:**
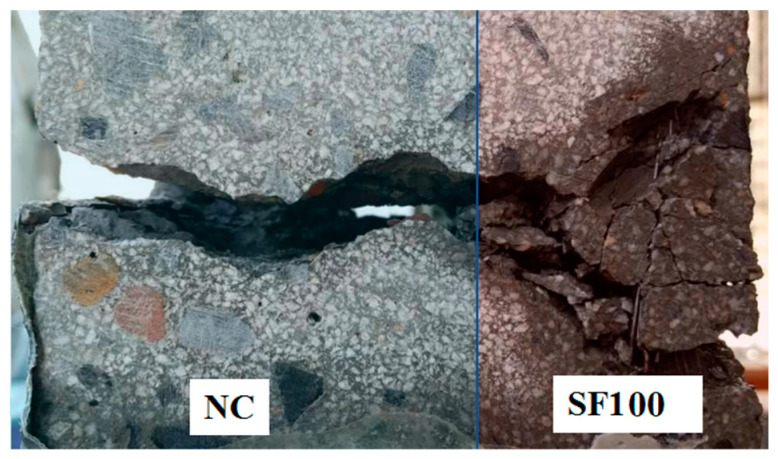
Crack pattern of the SF100-30% specimen.

Many fibers are distributed along the direction of stress on the specimen, and this results in the formation and propagation of multiple cracks in the FRC layer. In order to compare the morphology of cracks in the NC and FRC layers, the cracks of the specimens were enlarged, and the waterproof tape on the concrete surface was removed after the permeability test. Figure 14 takes the SF100-30% as an example and compares the morphology of cracks in the NC layer and the FRC layer in layered FRC specimens. It can be seen that the NC layer has only one crack with a large width, while the FRC layer has multiple cracks with smaller widths for each crack. According to the Poiseuille law, when the total width of concrete cracks remains constant, the greater the number of cracks, the higher the impermeability of the concrete.

Assuming that the surfaces of cracks are parallel, smooth, and conform to the Poiseuille law, as shown in Equation (2), when the total width of all cracks is 0.2 mm, and each crack in multiple cracks has an equal crack width, the following can be analyzed: when there is one crack, its width is 0.2 mm; when there are two cracks, each has a width of 0.2/2 mm, and so on. Table 12 compares the crack permeability of specimens with different numbers of cracks and the decreased rate of permeability of multiple cracks compared to one crack with a crack width of 0.2 mm.

According to Table 12, when the number of cracks is 2, 3, 4, and 5 compared to a single crack of 0.2 mm width, the crack permeability of specimens decreases by 75.0%, 88.8%, 93.8%, and 96.0%, respectively. Considering the effect of crack surface roughness on permeability, the surface area of cracks in the specimen with multiple cracks is significantly larger than that in the specimen with a single crack. Similar findings had already been reported: the smaller the crack width, the more significant the effect of the crack surface on crack permeability [38]. Therefore, compared to the crack permeability between single cracks and multiple cracks in concrete with the same crack width, the presence of multiple cracks can significantly decrease the crack permeability of concrete and enhance the impermeability of cracked concrete. 

From the analysis above, it can be seen that for layered FRC specimen structures, multiple cracks in the FRC layer and a rough crack surface considerably decrease the permeability of layered FRC specimens in concrete structures and prolong the service life of concrete structures. The study will provide theoretical support for the use of layered FRC to improve the impermeability of concrete structures in engineering applications such as pathways, tunnels, and other underground constructions.

## 4. Conclusions

This study investigated the effects of macro fibers on permeability and crack surface topography of layered FRC specimens with different layering ratios under uniaxial tensile load. The following conclusions can be drawn:

1. Under uniaxial tensile load, when the fiber volume content of FRC is equal, the tensile toughness of FRC is determined by the steel fiber proportion in macro fibers. The higher the proportion of steel fiber in macro fibers, the greater the average bearing capacity of FRC. The order of average bearing capacities for layered FRC specimens with the same layering ratio is: SF100 > SF80PP2.3 > SF20PP9.1 > PP11.4.
2. For layered FRC specimens, the crack permeability is much lower than that of the NC specimen. The SF80PP2.3 specimen demonstrates significantly good crack impermeability compared to mono FRC specimens (SF100 specimen and PP11.4 specimen). A significant positive synergistic effect on crack impermeability can be achieved by the combination of steel fiber and polypropylene fiber in the SF80PP2.3 specimen.
3. With the decreasing layering ratio, the crack surface *R*_n_ of layered FRC specimens gradually decreases. The crack surface *R*_n_ of the NC layer of layered FRC specimens (from 1.234 to 1.669) is all higher than that of the NC specimen (1.232), and it increases with the increase in both the layering ratio and the steel fiber proportion in macro fibers in the layered FRC specimens.
4. The crack surfaces in the FRC layer of layered FRC specimens (*R*_n_ from 1.806 to 2.170) are rougher than the crack surfaces in unlayered FRC specimens (*R*_n_ from 1.796 to 2.108). With the decreasing layering ratio, the roughness of crack surfaces in the FRC layer of layered FRC specimens increases.

## Figures and Tables

**Table 1:** Base mix proportion of concrete.

Cement (kg/m^3^)	Fly Ash (kg/m^3^)	Fine Aggregate (kg/m^3^)	Coarse Aggregate (kg/m^3^)	Water (kg/m^3^)	Superplasticizer (kg/m^3^)
390	155	822	848	272.5	8.0

**Table 2:** Parameters of macro fibers.

Fiber Type	Density (kg/m^3^)	Length (mm)	Diameter (mm)	Tensile Strength (MPa)	Elastic Modulus (GPa)	Number per Kilogram (piece/kg)	Number per Cubic Meter (piece/m^3^)
Polypropylene fiber	910	45	0.75	490	3.9	5.76 × 10^4^	5.24 × 10^7^
Steel fiber	7800	35	0.55	1150	200	1.45 × 10^4^	1.13 × 10^8^

**Table 3:** Fiber content of specimens.

Mixture ID	Steel Fiber	Polypropylene Fiber	Slump (mm)	Air Content (%)
SF100	100 kg/m^3^ (1.25 vol.%)	--	110	4.1
PP11.4	--	11.4 kg/m^3^ (1.25 vol.%)	156	3.0
SF80PP2.3	80 kg/m^3^ (1.0 vol.%)	2.3 kg/m^3^ (0.25 vol.%)	128	4.0
SF20PP9.1	20 kg/m^3^ (0.25 vol.%)	9.1 kg/m^3^ (1.0 vol.%)	140	3.1

**Table 4:** Comparison of uniaxial tensile properties of different layered FRC specimens.

Sample	F_P_	*P*_0.16_	*F*_0.16_	*P*_0.33_	*F*_0.33_
	(kN)	(kN)	(kN)	(kN)	(kN)
NC	34.9	--	--	--	--
SF100-100%	39.6	35.1	31.8	32.2	33.0
SF100-70%	34.3	31.7	28.8	30.1	29.9
SF100-50%	38.5	22.3	23.6	25.3	23.6
SF100-30%	37.0	11.3	13.2	12.3	12.7
PP11.4-100%	24.6	19.7	19.6	15.1	18.3
PP11.4-70%	28.5	15.0	15.6	14.4	15.0
PP11.4-50%	31.2	7.3	9	8.0	8.4
PP11.4-30%	29.1	1.2	2.4	1.2	2.5
SF80PP2.3-100%	36.5	30.4	26.4	31.3	29.0
SF80PP2.3-70%	29.5	25.8	23.6	23.8	24.0
SF80PP2.3-50%	31.8	22.1	21	18.6	20.5
SF80PP2.3-30%	37.4	12.5	13.8	8.7	11.9
SF20PP9.1-100%	32.9	27.5	25.6	22.1	24.6
SF20PP9.1-70%	28.0	24.6	23.8	15.7	21.2
SF20PP9.1-50%	32.1	17.1	16.2	16.7	16.7
SF20PP9.1-30%	32.5	4.6	6.8	5.0	5.8

**Table 5:** Decrease rate of uniaxial tensile parameters of layered FRC specimens compared to unlayered FRC specimens.

Sample	Decrease Rate				
	F_P_	*P*_0.16_	*F*_0.16_	*P*_0.33_	*F*_0.33_
SF100-100%	--	--	--	--	--
SF100-70%	13.4%	9.7%	9.4%	6.5%	9.4%
SF100-50%	−12.2%	29.7%	18.1%	15.9%	21.1%
SF100-30%	3.9%	49.3%	44.1%	51.4%	46.2%
PP11.4-100%	--	--	--	--	--
PP11.4-70%	−15.9%	23.9%	20.4%	4.6%	18.0%
PP11.4-50%	−9.5%	51.3%	42.3%	44.4%	44.0%
PP11.4-30%	6.7%	83.6%	73.3%	85.0%	70.2%
SF80PP2.3-100%	--	--	--	--	--
SF80PP2.3-70%	19.2%	15.1%	10.6%	24.0%	17.2%
SF80PP2.3-50%	−7.8%	14.3%	11.0%	21.8%	14.6%
SF80PP2.3-30%	−17.6%	43.4%	34.3%	53.2%	42.0%
SF20PP9.1-100%	--	--	--	--	--
SF20PP9.1-70%	14.9%	10.5%	7.0%	29.0%	13.8%
SF20PP9.1-50%	−14.6%	30.5%	31.9%	−6.4%	21.2%
SF20PP9.1-30%	−1.2%	73.1%	58.0%	70.1%	65.3%

**Table 6:** Comparison of the crack permeability of layered FRC specimens.

Sample	Crack Permeability (m^2^)		
	*κ_c-_*_100_	*κ_c-_*_200_	*κ_c-_*_300_
NC	2.52 × 10^−10^	1.75 × 10^−9^	5.15 × 10^−9^
SF100-100%	4.24 × 10^−12^	3.17 × 10^−11^	1.00 × 10^−10^
SF100-70%	8.58 × 10^−12^	5.68 × 10^−11^	1.66 × 10^−10^
SF100-50%	4.08 × 10^−11^	2.82 × 10^−10^	8.07 × 10^−10^
SF100-30%	6.96 × 10^−11^	4.16 × 10^−10^	1.28 × 10^−9^
PP11.4-100%	7.40 × 10^−11^	5.05 × 10^−10^	1.54 × 10^−9^
PP11.4-70%	8.71 × 10^−11^	6.78 × 10^−10^	2.43 × 10^−9^
PP11.4-50%	1.45 × 10^−10^	9.58 × 10^−10^	2.84 × 10^−9^
PP11.4-30%	2.13 × 10^−10^	1.40 × 10^−9^	4.28 × 10^−9^
SF80PP2.3-100%	6.24 × 10^−12^	3.78 × 10^−11^	1.23 × 10^−10^
SF80PP2.3-70%	5.91 × 10^−12^	4.57 × 10^−11^	1.34 × 10^−10^
SF80PP2.3-50%	1.26 × 10^−11^	9.32 × 10^−11^	2.87 × 10^−10^
SF80PP2.3-30%	4.25 × 10^−11^	3.17 × 10^−10^	8.41 × 10^−10^
SF20PP9.1-100%	1.35 × 10^−11^	1.16 × 10^−10^	3.24 × 10^−10^
SF20PP9.1-70%	4.55 × 10^−11^	3.00 × 10^−10^	9.50 × 10^−10^
SF20PP9.1-50%	6.42 × 10^−11^	7.01 × 10^−10^	1.83 × 10^−9^
SF20PP9.1-30%	1.10 × 10^−10^	7.07 × 10^−10^	2.28 × 10^−9^

**Table 7:** Comparison of crack permeability parameters *ɑ* of the specimens.

Layering Ratio	Crack Permeability Parameter *ɑ*				
	NC	SF100	PP11.4	SF80PP2.3	SF20PP9.1
100%	12,108.3	224.9	3553.9	281.4	748.8
70%	--	396.6	5128.7	311.8	1881.3
50%	--	1924.2	6685.0	658.3	4464.1
30%	--	2951.1	9895.1	2038.3	5205.4

**Table 8:** Comparison of crack surface *R*_n_ of layered FRC specimens.

Sample	Roughness Parameter of Crack Surface, *R*_n_		
	*R*_n_ of NC Layer	*R*_n_ of FRC Layer	*R*_n_ of Entire Crack Surface
NC	1.232	--	1.232
SF100-100%	--	2.108	2.108
SF100-70%	1.614	2.137	1.980
SF100-50%	1.424	2.136	1.780
SF100-30%	1.304	2.170	1.564
PP11.4-100%	--	1.796	1.796
PP11.4-70%	1.311	1.806	1.658
PP11.4-50%	1.234	1.826	1.530
PP11.4-30%	1.242	1.862	1.428
SF80PP2.3-100%	--	2.016	2.016
SF80PP2.3-70%	1.669	2.072	1.951
SF80PP2.3-50%	1.535	2.062	1.799
SF80PP2.3-30%	1.354	2.132	1.587
SF20PP9.1-100%	--	1.902	1.902
SF20PP9.1-70%	1.461	1.895	1.765
SF20PP9.1-50%	1.335	1.902	1.619
SF20PP9.1-30%	1.246	1.932	1.452

**Table 9:** Decrease rate of *R*_n_ of layered FRC specimens compared to unlayered FRC specimens.

Layering Ratio	SF100	PP11.4	SF80PP2.3	SF20PP9.1
100%	--	--	--	--
70%	6.1%	7.7%	3.2%	7.2%
50%	15.6%	14.8%	10.8%	14.9%
30%	25.8%	20.5%	21.3%	23.7%

**Table 10:** Increase rate of crack surface *R*_n_ of the NC layer in layered FRC compared to the NC specimen.

Layering Ratio	SF100	PP11.4	SF80PP2.3	SF20PP9.1
70%	31.0%	6.4%	35.5%	18.6%
50%	15.6%	0.2%	24.6%	8.4%
30%	5.8%	0.8%	9.9%	1.1%

**Table 11:** Increase rate of crack surface *R*_n_ of the FRC layer in layered concrete compared to unlayered FRC specimens.

Layering Ratio	SF100	PP11.4	SF80PP2.3	SF20PP9.1
70%	1.4%	0.6%	2.8%	−0.4%
50%	1.3%	1.7%	2.3%	0.0%
30%	2.9%	3.7%	5.8%	1.6%

**Table 12:** Comparison of multiple crack permeabilities according to the Poiseuille law.

Number of Cracks	Width of Single Crack (mm)	Permeability of One Crack (m^2^)	Permeability of All Cracks (m^2^)	Percentage Reduction of the Crack Permeability Compared to One Crack of 0.2 mm Width
1	0.2	3.33 × 10^−9^	3.33 × 10^−9^	0
2	0.1	8.33 × 10^−10^	1.67 × 10^−9^	75.0%
3	0.067	3.74 × 10^−10^	1.12 × 10^−9^	88.8%
4	0.050	2.08 × 10^−10^	8.33 × 10^−10^	93.8%
5	0.040	1.33 × 10^−10^	6.67 × 10^−10^	96.0%

## Data Availability

Data available on request from the authors. The data that support the findings of this study are available from the corresponding author upon reasonable request.
